# Integrated whole liver histologic analysis of the allogeneic islet distribution and characteristics in a nonhuman primate model

**DOI:** 10.1038/s41598-020-57701-8

**Published:** 2020-01-21

**Authors:** Geun Soo Kim, Jong Hyun Lee, Du Yeon Shin, Han Sin Lee, Hyojun Park, Kyo Won Lee, Heung-Mo Yang, Sung Joo Kim, Jae Berm Park

**Affiliations:** 1 Samsung Advanced Institute for Health Sciences & Technology, Graduate School, Department of Health Sciences & Technology, Sungkyunkwan University, Seoul, Republic of Korea; 2 Stem Cell & Regenerative Medicine Institute, Samsung Medical Center, Seoul, Republic of Korea; 3 Transplantation Research Center, Samsung Medical Center, Seoul, Republic of Korea; 4 Department of Surgery, Samsung Medical Center, Sungkyunkwan University School of Medicine, Seoul, Republic of Korea; 5 Division of Endocrinology and Metabolism, Department of Medicine, Samsung Medical Center, Sungkyunkwan University School of Medicine, Seoul, Republic of Korea; 6 Department of Medicine, Sungkyunkwan University School of Medicine, Gyeonggi, Republic of Korea; 7 GenNBio Inc, Seoul, Republic of Korea

**Keywords:** Endocrinology, Immunohistochemistry

## Abstract

The most obvious method to observe transplanted islets in the liver is direct biopsy, but the distribution and location of the best biopsy site in the recipient’s liver are poorly understood. Islets transplanted into the whole liver of five diabetic cynomolgus monkeys that underwent insulin-independent survival for an extended period of time after allo-islet transplantation were analyzed for characteristics and distribution tendency. The liver was divided into segments (S1–S8), and immunohistochemistry analysis was performed to estimate the diameter, beta cell area, and islet location. Islets were more distributed in S2 depending on tissue size; however, the number of islets per tissue size was high in S1 and S8. Statistical analysis revealed that the characteristics of islets in S1 and S8 were relatively similar to other segments despite various transplanted islet dosages and survival times. In conclusion, S1, which exhibited high islet density and reflected the overall characteristics of transplanted islets, can be considered to be a reasonable candidate for a liver biopsy site in this monkey model. The findings obtained from the five monkey livers with similar anatomical features to human liver can be used as a reference for monitoring transplanted islets after clinical islet transplantation.

## Introduction

The transplantation of pancreatic islets into the liver is one method for the treatment of type 1 diabetes. In the past, maintenance of normal glucose levels without exogenous insulin after transplantation was relatively short in duration^1–3^. However, improvements lasting more than 5 years have recently been reported^4^.

To date, clinical islet transplantation and isolation of islets have made remarkable progress; however, problems remain. Therefore, many research groups around the world use nonhuman primates (NHPs) as experimental animals for preclinical studies involving pancreatic islet transplantation. The NHP model is an adequate representation of human physiological characteristics and the immune system compared with other experimental animals^5–7^. Thus, NHPs are widely used to study various diseases as well as newly developed antibodies or drugs^8–11^.

In islet transplantation studies, observing the transplanted islets in the recipient liver is very important because islets that have achieved insulin-independent normal glucose levels under various conditions during the early and extended periods after transplantation provide meaningful information that can aid progress in the islet transplantation field^12–16^. The most obvious method to observe islets transplanted into the recipient liver and surrounding environment is direct liver biopsy. Liver biopsy has been used as a method for monitoring transplanted islets since the first liver biopsy was performed in islet allotransplanted patients in 1991^17^. However, there are a number of limitations, such as pain, infection, bleeding, and postbiopsy management, for patients undergoing liver biopsy^18^. In addition, the probability of finding the transplanted islets in the obtained tissue is not high^19^. Therefore, noninvasive islet follow-up studies using magnetic resonance imaging (MRI) or computed tomography (CT) have been extensively performed and demonstrate the importance and risks of indirect liver biopsy^20–28^.

Currently, descriptions regarding the characteristics of transplanted islets such as islet distribution, size, beta cell mass, and transplanted location at the whole liver level have been poorly detailed in islet transplantation and related studies. In previous studies involving rodents^29,30^, pigs^31^, and primates^32^, features of transplanted islets have only been identified in parts of the liver. Because the liver is generally divided into a number of lobes, the characteristics of the islets found in one or several regions do not necessarily reflect the characteristics of the transplanted islets in the whole liver. Thus, finding the site with the highest density of islets in the liver will have a beneficial effect on the successful detection of islets and reduce the risk associated with liver biopsy.

In the present study, the distribution and characteristics of transplanted islets in NHP whole liver were observed, and a suitable site for liver biopsy was suggested. The distribution and characteristics of islets analyzed in the whole liver of five diabetic cynomolgus monkeys that experienced insulin-independent survival for an extended period after allo-islet transplantation are reported.

## Results

### Graft function after islet transplantation

**Figure 1:**
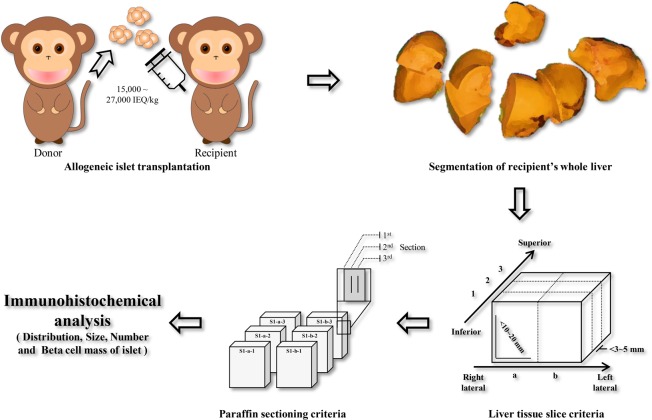
Whole liver mapping (LM) process. Diabetic recipient monkeys received islets isolated from donor monkeys through portal vein infusion. After transplantation, the whole liver of the monkey that achieved normal blood glucose levels without exogenous insulin was prepared and divided into segments. The divided liver segments were sliced with uniform size and order based on anatomic features of the monkey. Paraffin slides were produced from the uniformly sliced tissue for immunohistochemistry analysis.

As shown in Fig. 1, the overall analysis of allogeneic islet grafts was performed using a diabetic cynomolgus monkey model. First, diabetic monkeys were transplanted with 15,000 to 27,000 islet equivalents (IEQ)/kg body weight after islet isolation (Fig. 1). A different amount of islets (IEQ) was transplanted into each monkey to determine whether the islet distribution was constant depending on the number of transplanted islets (Table 1). After islet transplantation, islet graft function was monitored based on the duration of normal glucose and serum C-peptide levels maintained without exogenous insulin (Supplemental Fig. S1). The average period of normoglycemia was 87.2 ± 22.8 days (Table 1). LM-1 (POD 33) and LM-3 (POD 54) were terminated at an early time point due to infection. LM-2 was terminated at an early time point due to technical failure (POD 77). LM-4 (POD 163) and LM-5 (POD 109) completed the experiment according to our schedule. Blood glucose levels of early terminated monkeys were in the normal range and did not show changes in significant health status at those times. In addition, all five monkeys increased or maintained their body weight during the normoglycemia period. Insulin staining performed on the necropsied pancreatic tissues of the monkeys (LM-1, LM-2, LM-4, and LM-5) at the end point of the experiment indicated that all of the endogenous islets of the monkeys were destroyed (Supplementary Fig. S2). Pancreas tissue could not be taken from LM-3 due to adhesions, along with the extremely low amount of pancreatic tissue. This result indicated that monkeys achieved normal blood glucose level maintenance with transplanted islets only. Therefore, the transplanted allogeneic islet graft was well tolerated in the liver tissue of recipient monkeys.

**Table 1:** Characteristics of the five cynomolgus monkeys.

	LM-1	LM-2	LM-3	LM-4	LM-5
POD	33	77	54	163	109
Sex	M				
Age (months)	63	51	72	51	51
Body weight (kg)	3.8	3.16	3.5	3.6	3.4
Transplanted islet dose (IEQ/kg)	15,000	21,000	20,000	26,882	27,000
Counted islet number after LM (islets)	308	695	1,893	850	1,199

LM, liver mapping; POD, postoperative date; IEQ, islet equivalent

### Whole liver tissue processing

**Figure 2:**
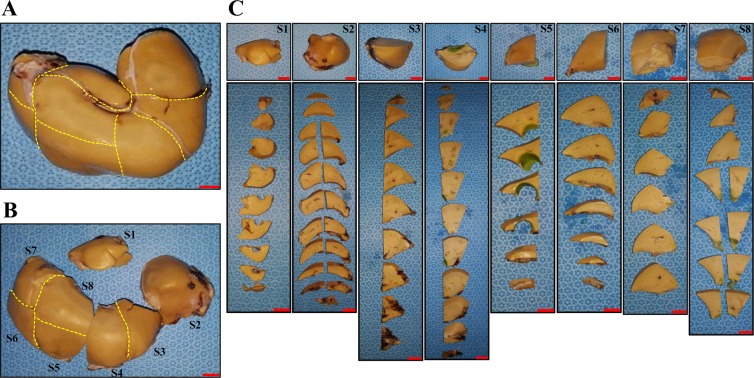
Segmentation of the whole liver. Representative whole liver of monkey LM-5. (**a**) Anterior view of the whole liver. (**b**) The formalin-fixed whole liver was divided into eight segments. (**c**) Divided liver tissues were evenly sliced using liver tissue slice criteria (scale bars = 1 cm).

Five whole livers were fixed with 10% NBF solution for 72 hours to obtain sufficient fixation and divided into segments S1–S8 according to the cynomolgus monkey liver anatomy^33^. The whole liver tissue of the recipient monkey was sliced, and paraffin sectioning was performed following our established protocols (Fig. 1). Consequently, 449 tissue blocks (107 from LM-1, 93 from LM-2, 93 from LM-3, 78 from LM-4, and 78 from LM-5) (Fig. 2) and 9,735 paraffin section slides (3,210 from LM-1, 1,395 from LM-2, 2,790 from LM-3, 1,170 from LM-4, and 1,170 from LM-5) were produced.

### Two-dimensional mapping of whole liver

**Figure 3:**
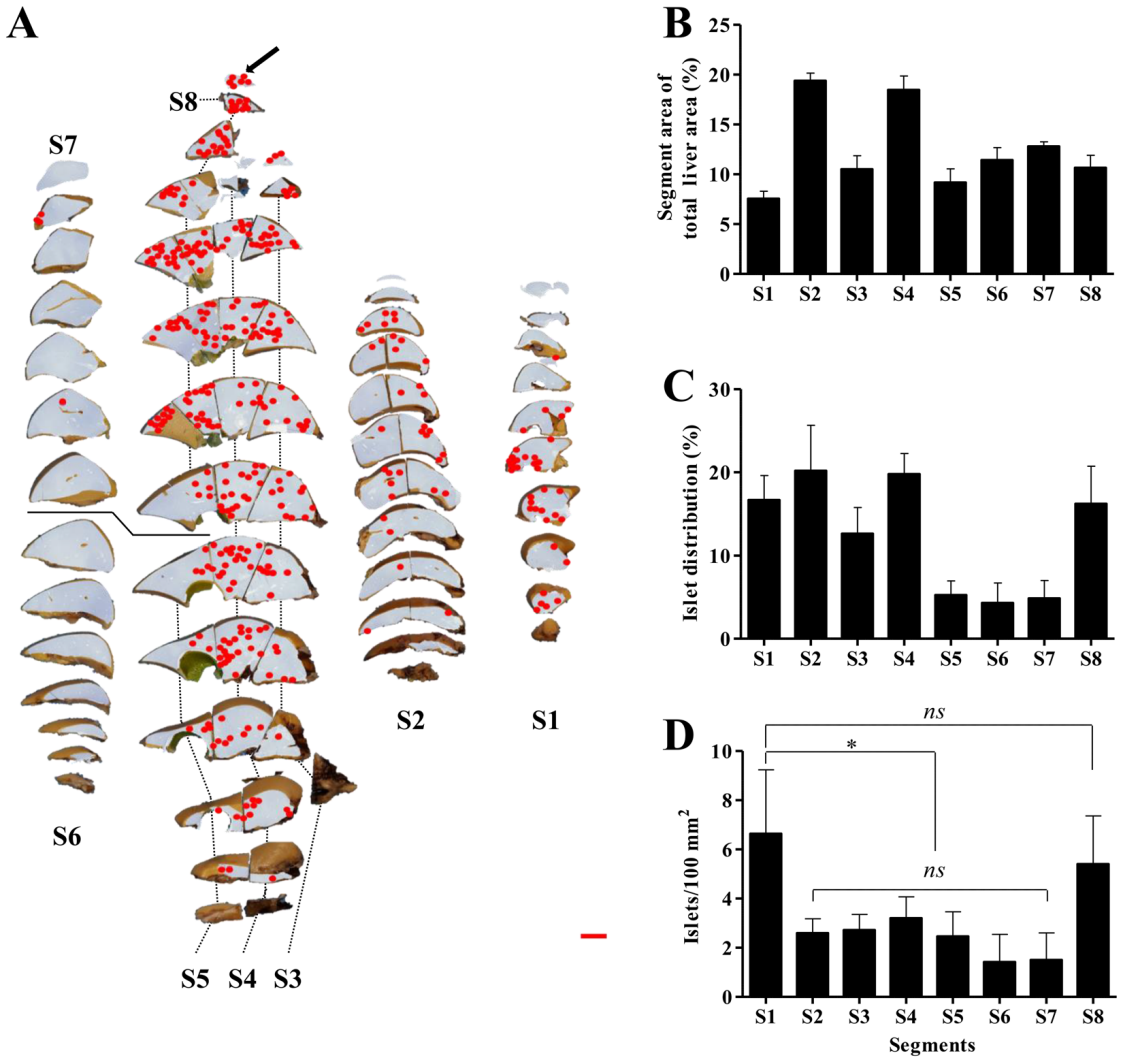
Two-dimensional mapping results of the recipient monkey’s whole liver (LM-5) and distribution of transplanted islets. (**a**) Sliced liver tissues overlapped with insulin-stained liver tissue images reassembled into the same shape as before slicing. Transplanted islets in tissue are indicated with dots (arrowed) (scale = bar, 1 cm). Analyzed (**b**) liver tissue area of each segment, (**c**) islet distribution of each segment and (**d**) islet number per tissue area (100/mm^2^) were obtained after transplanted islet counting and analysis using the Positive Pixel Count algorithm v9.1 *p < 0.05, *ns* > 0.05. The data are depicted as the means ± SEM (*n* = 5).

For accurate counting of islets grafted to the liver tissue, the following criteria were established: (1) coexpression of insulin and the pancreatic islet nucleus, and (2) islet diameter > 50 μm (Supplemental Fig. S3a). After immunohistochemistry staining, circles indicating islets were marked on analyzed liver tissue images for visualization (Supplemental Fig. S3b), and these images were reconstructed into a two-dimensional liver image as observed before sectioning. Whole liver tissue was successfully recombined, and grafted islets could be quickly visualized in the whole liver (Fig. 3A).

### Area of liver tissue

The area of analyzed liver tissue and the number of counted islets are presented as percentages after considering individual differences (Fig. 3B,C). The analyzed segment areas of the whole livers of the five monkeys were 7.6 ± 0.1% (S1), 19.4 ± 0.8% (S2), 10.5 ± 1.4% (S3), 18.5 ± 1.4% (S4), 9.2 ± 1.4% (S5), 11.4 ± 1.3% (S6), 12.8 ± 0.5% (S7), and 10.7 ± 1.2% (S8). Based on the results, the area of the median lobe (S3, S4, S5, and S8) was the largest, and the right lateral lobe (S6 and S7) and left lateral lobe (S2) were the next largest. Conversely, the caudate lobe (S1) had the smallest area. Regarding single segment areas, S2 and S4 were the largest, and S1 was the smallest (Fig. 3B).

### Distribution of allogeneic grafted islets in the liver tissue

To verify the distribution of allogeneic islets after transplantation, immunohistochemistry staining for insulin was performed on 1,347 slides, and 4,945 islets were found (308 from LM-1, 695 from LM-2, 1,893 from LM-3, 850 from LM-4, and 1,199 from LM-5). The islets were less distributed in S5 (5.3 ± 1.7%), S6 (4.3 ± 2.4%), and S7 (4.9 ± 2.2%). The islets were more distributed in S2 (20.2 ± 5.5%) and S4 (19.8 ± 2.4%) depending on the tissue area. The islets were more distributed in S1 (16.7 ± 3.0%), S3 (12.6 ± 3.2%), and S8 (16.2 ± 4.5%) than in S5, S6, and S7 (Fig. 3C). However, the number of islets per liver tissue area (100 mm^2^) was the highest in S1 (6.6 ± 2.6 islets) and S8 (5.4 ± 2.0 islets) (Fig. 3D). These results indicate that the allogeneic grafted islets were distributed with the highest density in S1 and S8.

### Characteristics of allogeneic grafted islets in the liver tissue

**Figure 4:**
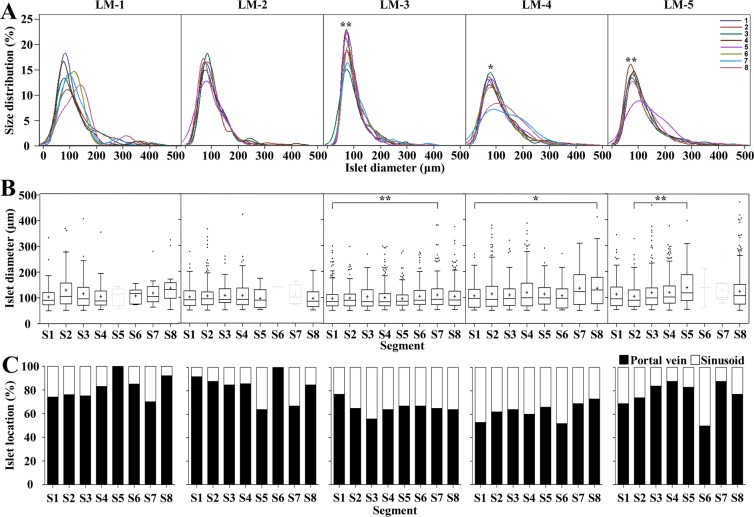
The characteristics of transplanted islets in five monkey livers were similar in whole segments. Diameters of islets and of grafted islets were obtained after analysis using the Positive Pixel Count algorithm. (**a**) Linear graphs represent the overall similarity of segments between monkeys. Each segment was labeled with a different color line. The y axis indicates the distribution of the counted islets’ diameter. *p < 0.05, **p < 0.01. (**b**) The statistical similarity of the column graph indicates the similarity of each segment to S1 or S8. Segments with less than 10 islets are shown in light gray and were excluded from statistical analysis. The diameter of the counted islets showed similarity in whole segments with the exception of monkey LM-3. *p < 0.05, **p < 0.01. (**c**) The location of grafted islets was determined after islet counting. Islets located in the grafted site of the portal triad zone and sinusoid were designated as the portal vein and sinusoid, respectively. Transplanted islets were highly distributed in the portal vein site.

Next, whether the characteristics of the islets found in S1 and S8 were similar to the other segments was investigated. First, the size of the transplanted islets in each segment was analyzed using statistical methods to observe similarities among the five monkeys. Due to the nonuniform shape of the islets, the islet diameter was determined using the average of the two values obtained from two common formulas for the diameter of a circle^34^. The mean islet diameter was the greatest in S7 (113.7 ± 3.2 μm) and S8 (115.8 ± 2.0 μm). In statistical analysis, no differences were found between the whole segments in monkeys LM-1 and LM-2 (Fig. 4A). However, no differences were observed between the remaining segments to S1 or S8 in monkeys LM-4 and LM-5. However, a difference was observed between S1 and S7 from LM-3 (Fig. 4B). Next, the function of grafted islets was analyzed based on the insulin-positive area (beta cell area) within the total islet area. The beta cell area of grafted islets was greatest in S1 (50.6 ± 0.6%) and S2 (49.2 ± 0.6%). However, statistical similarity, such as islet diameter, was not observed (Supplemental Fig. S4). Lastly, the location of grafted islets was analyzed. Islets located in the portal triad zone and sinusoid were designated as portal veins and sinusoids, respectively (Supplemental Fig. S3d). The location of islets in each segment of the five monkeys was not as similar as the diameter or beta cell area. However, the islets were more distributed in the portal vein than in the sinusoid (Fig. 4C). The data indicated that the characteristics of transplanted islets in S1 and S8 were similar to other segments in the five monkeys, and specifically, the size of the islets that could affect the success rate of the liver biopsy was statistically similar.

## Discussion

The liver is mainly considered a suitable site for islet transplantation^16^. Islets transplanted into the portal vein are considered transplanted into the entire liver *via* the bloodstream. However, to the best of our knowledge, no reports have been published on the distribution or characteristics of transplanted islets in the whole liver of cynomolgus monkeys. The liver tissues and islets were labeled with unique identification names based on our criteria described in the Methods. The results obtained by this classification criterion can be variably applied even when using different criteria distinct from our anatomical explanation of the cynomolgus monkey liver applied in this study^33^.

The number of counted islets was lower in S5, S6, and S7 and higher in S2, S3, and S4 depending on the tissue area (Fig. 3C). However, the islet number per liver tissue area (100 mm^2^) was highest in S1 and S8 (Fig. 3D). S1 had the smallest tissue area but had the highest islet number per liver area. S1 is considered to contain a high density of islets in terms of the first encounter of transplanted islets through the portal vein and an independent supply of blood from the main portal vein. However, why S8 had the highest distribution of islets is difficult to explain. This phenomenon was determined to be caused by differences in blood flow attributed to the distinction between the posterior right portal vein (S6, S7) with its acute angle and the anterior right portal vein (S8) with its obtuse angle. These results are in contrast to the results from studies in which the distribution of islets transplanted from rhesus monkeys was uniform in all segments and the smaller-sized islets were transplanted into caudate lobes in rodents^29,32^. In addition, evaluation of the effects of embolization based on islet transplantation in pigs showed that the distribution of microspheres representing islets injected per tissue weight was highest in S6 and S7, which was in contrast with the results from the present study^31^. However, the difference in liver anatomical structure between primates and other laboratory animals should be noted. As the dose of transplanted islets increased, the results from the distribution of islets at a higher density in S8 ( > 26,000 IEQ/kg) indicated that further studies on a liver that receives a high dose of islets are needed. In addition, the study of the distribution and number of islets in the condition of maintaining normal blood glucose without exogenous insulin for a long period of one year or more after transplantation is necessary.

For many years, researchers have been interested in the relationship between islet size and transplantation outcomes^30,35–40^. Small islets have a higher expression of factors involved in angiogenesis than large islets and may have a positive effect on transplant outcome due to reduced embolism based on the results of islets transplanted in small rodents^30,36^. Islets experience a hypoxic environment immediately after isolation from the pancreas and a hypoxic state due to delayed vascularization caused by a lack of oxygen supply by the thrombosis on the surface after being transplanted into the liver^37^. Specifically, the hypoxic environment inside the islets increases with larger islets, leading to apoptosis and resulting in poor viability, function, and consequently, negative effects on islet transplantation prognosis^38,39^. Therefore, in the present study using cynomolgus monkeys, the results would hypothetically show a higher proportion of smaller islets. As expected, the counted islets were mostly between 50 and 100 μm in diameter, and the number decreased with increasing size (Supplemental Fig. S5a). The results, which showed a significantly larger distribution of small islets in the recipient liver with good prognosis after transplantation, are consistent with previous reports that small islets contributed to the transplantation outcome and show that this phenomenon is similar in cynomolgus monkeys^36,40^.

Similar to the correlation between the islet size and the results of transplantation, in many studies, the ratio of insulin-expressing beta cell content of islets was higher in smaller islets^36,41–43^. The results from the present study confirmed this finding. We decided that the most significant contributor to the normalization of glucose levels in islets transplanted into diabetic monkeys is beta cells in islet clusters that express insulin. As expected, beta cell expression in the five monkeys ranged from 23.7 ± 7.4% to 52.7 ± 1.0%, and the expression level was higher in smaller islets (Supplemental Fig. S5b). The results from the present study are consistent with previous theories stating that islet size correlates with the expression ratio of beta cells and is related to function^38,41,43^.

Regarding the relationship between the size and function of islets and the success rate of transplantation, the location of the grafted islets should also be considered. Laboratory rodents have a smaller liver vascularity than humans; however, the mean size of the islets after isolation is larger and can cause severe embolism in the early stage of allogeneic transplantation through the portal vein^22,29,30,44^. Most islets transplanted through the portal vein in human^12,13,19,43,45,46^ or primate^32,47–52^ livers were found in the portal vein or portal triad zone. However, the data for transplanted islet location at the whole liver level are insufficient. Based on the results from the present study, unlike the size and insulin expression ratio of islets, the location of the transplanted islets in our five monkeys was not consistent but showed the portal vein at a rate of 60% or more except for a few segments (LM3-S3, LM4-S1-S6, and LM5-S6). In addition, the islets transplanted into the portal vein and sinusoid were classified based on size. Many small islets (<100 μm in diameter) were localized in the sinusoid, and larger islets (>300 µm in diameter) were not found in the sinusoid (Supplemental Fig. S5c). These results were predictable based on a number of published studies; however, it is interesting that the characteristics of the analyzed islets of the five monkeys were similar in all segments despite the difference in the amount of transplanted islets and the survival time.

In conclusion, S1, which exhibited a high islet density and reflected the overall characteristics of islets transplanted into the liver, can be considered a candidate for a highly successful liver biopsy site in cynomolgus monkeys. Although the primate liver used in this study does not exactly represent the human liver, the information obtained can be used as a reference for monitoring transplanted islets after clinical islet transplantation. The results from the present study provide a reasonable biopsy site for monitoring islets more accurately in primate animal models used for various islet transplantation studies and potential clinical applications.

## Materials and Methods

### Animals and animal care

Cynomolgus monkeys (*Macaca fascicularis*) were supplied by Orient Bio Co. Ltd (Seongnam, Korea). The average weight and age of the five male monkeys were 3.5 ± 0.1 kg and 57.6 ± 4.3 months, respectively. All procedures related to infection screening, housing, handling, care and treatment in this study were performed as previously described^53^. The study was approved by the Institutional Animal Care and Use Committee of Orient Bio Laboratories (ORIENT-IACUC-13001) and the experiments were performed in accordance with the relevant guidelines and regulations.

### Induction of diabetes mellitus in cynomolgus monkeys

All procedures for the induction, confirmation, and maintenance of type 1 diabetes in cynomolgus monkeys were performed as previously described^53^. Briefly, diabetes was induced by injecting 60 mg/kg streptozotocin (Sigma, St. Louis, MO, USA) after surgical removal of >70% of the recipient monkey’s pancreas. Confirmation of diabetes was based on the following three criteria: (1) blood glucose level (>250 mg/dL), (2) fasting C-peptide level < 0.5 ng/mL, and (3) the absence of stimulated C-peptide and endogenous insulin response based on the intravenous glucose tolerance test (IVGTT). After successful induction of diabetes and islet transplantation, blood glucose levels were checked 3 to 4 times daily, and exogenous insulin (glargine: Lantus; Sanofi-Aventis, Bridgewater, NJ, USA, and glulisine: Apidra, Sanofi-Aventis) was used to maintain blood glucose levels < 200 mg/kg.

### Islet isolation

Islet isolation and transplantation were performed as previously described^53^. Briefly, islets were isolated from monkey pancreas using the modified Ricordi method with collagenase MTF C/T (Roche, Mannheim, Germany). For the purification of isolated islets, the discontinuous Ficoll density gradient method was used. Next, purified islets were cultured with CMRL-1066 medium (Corning, NY, USA) containing 10% fetal bovine serum (FBS; Gibco-Thermo Fisher Scientific, Waltham, MA, USA) and 1% antibiotics (Gibco-Thermo Fisher Scientific) in a humidified 5% CO_2_ atmosphere at 37 °C.

### Immunosuppression and islet transplantation

Cultured islets were transplanted into the portal veins of five monkeys as previously described^54^ (Fig. 1). Briefly, under general anesthesia, a tunneled implantable venous access port was inserted via the right internal jugular vein for drug and fluid delivery. A dose of 5 mg/kg rabbit anti-thymocyte globulin (rATG, Genzyme, Cambridge, MA) was administered in combination with 20 mg/kg hydrocortisone and 2 mg of pheniramine to prevent hypersensitivity reactions against immunosuppressants. All monkeys received rATG 4 times over 12-hour intervals up to a cumulative dose of 20 mg/kg to induce immunosuppression. After laparotomy, the portal vein was isolated, and an 18-gauge angiocatheter was inserted. Islets mixed with heparin were slowly infused through the angiocatheter into the portal vein. After the islet infusion, the angiocatheter was removed, and the puncture hole was closed with sutures. The laparotomy was closed, and the monkey was returned to its cage after fully awakening from anesthesia.

### Perioperative management

Perioperative management was performed as previously described^54^. Briefly, to maintain the immunosuppression regimen, tacrolimus (FK506) with a target trough level of 5–10 mg/ml and mycophenolate mofetil (MMF) with a target trough level of 1–3 mg/ml were orally administered. Tramadol and cefazolin were used to control surgical site pain and prophylactic antibiotic use. Ganciclovir was administered for prophylaxis against CMV reactivation. Blood was drawn two times per week for two weeks and once per week thereafter for hematologic and serum chemistry tests. The C-peptide level of serum was measured using a radioimmunoassay kit (C-peptide IRMA kit; IMMUNOTHECH, Beckman Coulter, Prague, Czech Republic) according to the manufacturer’s instructions.

### Preparation of whole liver and pancreas tissue

Whole liver tissues were obtained from five monkeys at the end of the study. Whole liver tissues were fixed with 10% normal buffered formalin (NBF) solution for 72 hours and divided into eight segments (S1–S8) as previously described^33^. Next, each segment was uniformly sliced into sections 3–5 mm thick and 10–20 mm wide. Each tissue was organized according to our own labeling method. Based on the anatomic feature of the monkey, the inferior to superior direction of each segment of the liver was labeled with a number, and the right lateral to left lateral direction was labeled with a letter (Fig. 1). Pancreas tissues were obtained from monkeys at the end of the study to confirm that all the endogenous islets of the original monkeys had been destroyed.

### Slide preparation and immunohistochemistry staining of islet-grafted whole liver tissue

Slides were prepared from paraffin-embedded liver tissue slices (3–5 mm in thickness) as described below. After initially trimming approximately 500 μm of liver tissue, 5–10 serial slides 4 μm in thickness were obtained. Then, the same method was repeated twice, resulting in three groups of 5–10 serial slides with an average of 15 to 30 slides per one slice of liver tissue (Fig. 1). Next, to investigate the immunohistochemical allogeneic islet graft, hematoxylin and eosin (H&E) and insulin staining were performed with the first and second slides from each group. For insulin staining, each slide was stained with rabbit anti-insulin antibody (Santa Cruz, TX, USA). Then, 3,3′-diaminobenzidine tetrahydrochloride (DAB) staining was performed using the DAKO EnVision system (DAKO, Santa Clara, CA, USA) according to the manufacturer’s instructions.

### Image acquisition

Whole slide images of both H&E slides and insulin immunohistochemistry slides were acquired by scanning the original glass slides using the ScanScope AT slide scanner (Leica Biosystems, Wetzlar, Germany) with a 20x lens. The default autofocus mode was used; however, in a few cases, manual focus was applied to optimize sharpness. The image files were stored using Aperio ScanScope software (Leica Biosystems).

### Image analysis

Using the Aperio ImageScope program (version 12.1.0.5029; Leica Biosystems), the area of analyzed liver tissue and boundaries of insulin-positive islet clusters were determined. Then, the islet diameter (μm), islet area (μm^2^), insulin-positive area within the total islet area (%), and analyzed liver tissue area (μm^2^) were obtained using the Aperio Positive Pixel Count algorithm (version 9.1).

### Statistical analysis

Statistical analysis of the grafted islet number was performed using the Wilcoxon matched-pairs signed rank test (one-tailed) (Fig. 3D). The islet diameter and beta cell area within the islets in the livers of the five monkeys were analyzed using one-way ANOVA with Bonferroni’s multiple comparison post hoc test with SAS version 9.4 (SAS Institute Inc, Cary, NC, USA) and GraphPad Prism version 5.00 (GraphPad software, San Diego CA, USA) (Fig. 4A,B, Supplemental Figs. S2f and S4a,b). To increase the reliability of the statistical analyses, segments with less than 10 islets were excluded from the analysis based on liver mapping of monkeys 1–5 (LM-1–LM-5) as follows: LM-1-S5, LM-2-S6-S7, and LM-5-S6-S7 (Fig. 4B). All data are presented as the means ± standard error of the mean (SEM).

## Supplementary information

Supplementary information.

## Data Availability

The datasets generated during and/or analyzed for the current study are available from the corresponding author on reasonable request.
