# Supplementary material for: Integrated whole liver histologic analysis of the allogeneic islet distribution and characteristics in a nonhuman primate model
Source: Sci Rep. 2020 Jan 21;10:793. doi: 10.1038/s41598-020-57701-8 (PMC6972963; doi:10.1038/s41598-020-57701-8)
Supplement: Supplementary file 1 — Supplementary information. [file 41598_2020_57701_MOESM1_ESM.pdf]

Supplementary information

Integrated whole liver histologic analysis of the allogeneic islet distribution and characteristics in a nonhuman primate model

Geun Soo Kim,[1,2,3] Jong Hyun Lee,[7] Du Yeon Shin,[1,2,3] Han Sin Lee,[2,5] Hyojun Park,[6,7] Kyo Won Lee,[4,6] Heung-Mo Yang, [6,7] Sung Joo Kim,[6,7] and Jae Berm Park,[1,2,3,4,6]

Supplemental figure 1.

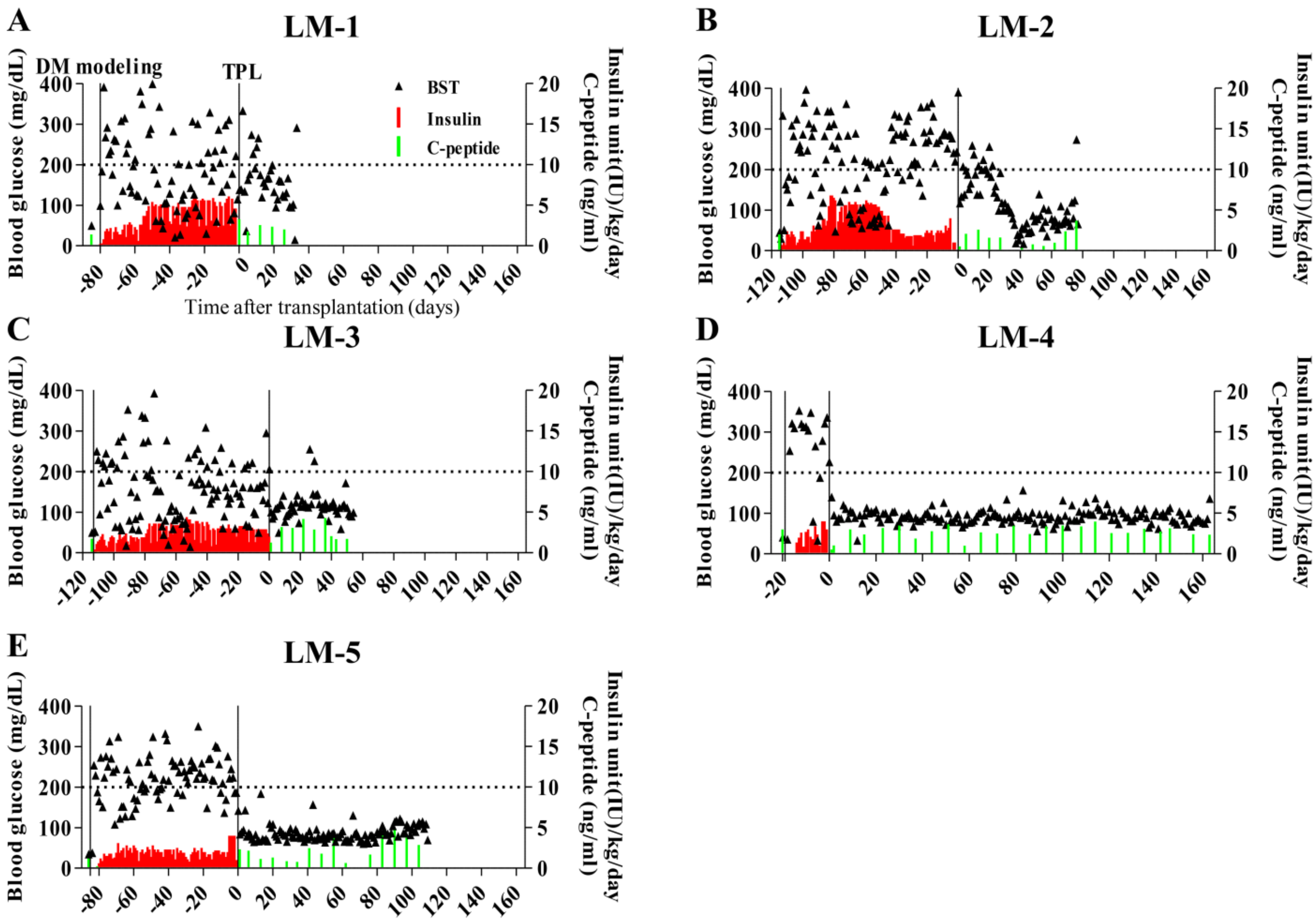

Supplemental Figure S1. Islet transplantation results in five monkeys. Five islet-infused monkeys were monitored for fasting blood glucose, serum C-peptide and insulin administration levels. (**a**) LM-1, (**b**) LM-2, (**c**) LM-3, (**d**) LM-4, and (**e**) LM-5. BST, blood sugar test; LM, liver mapping; TPL, transplantation; DM, diabetes modeling.

Supplemental figure 2.

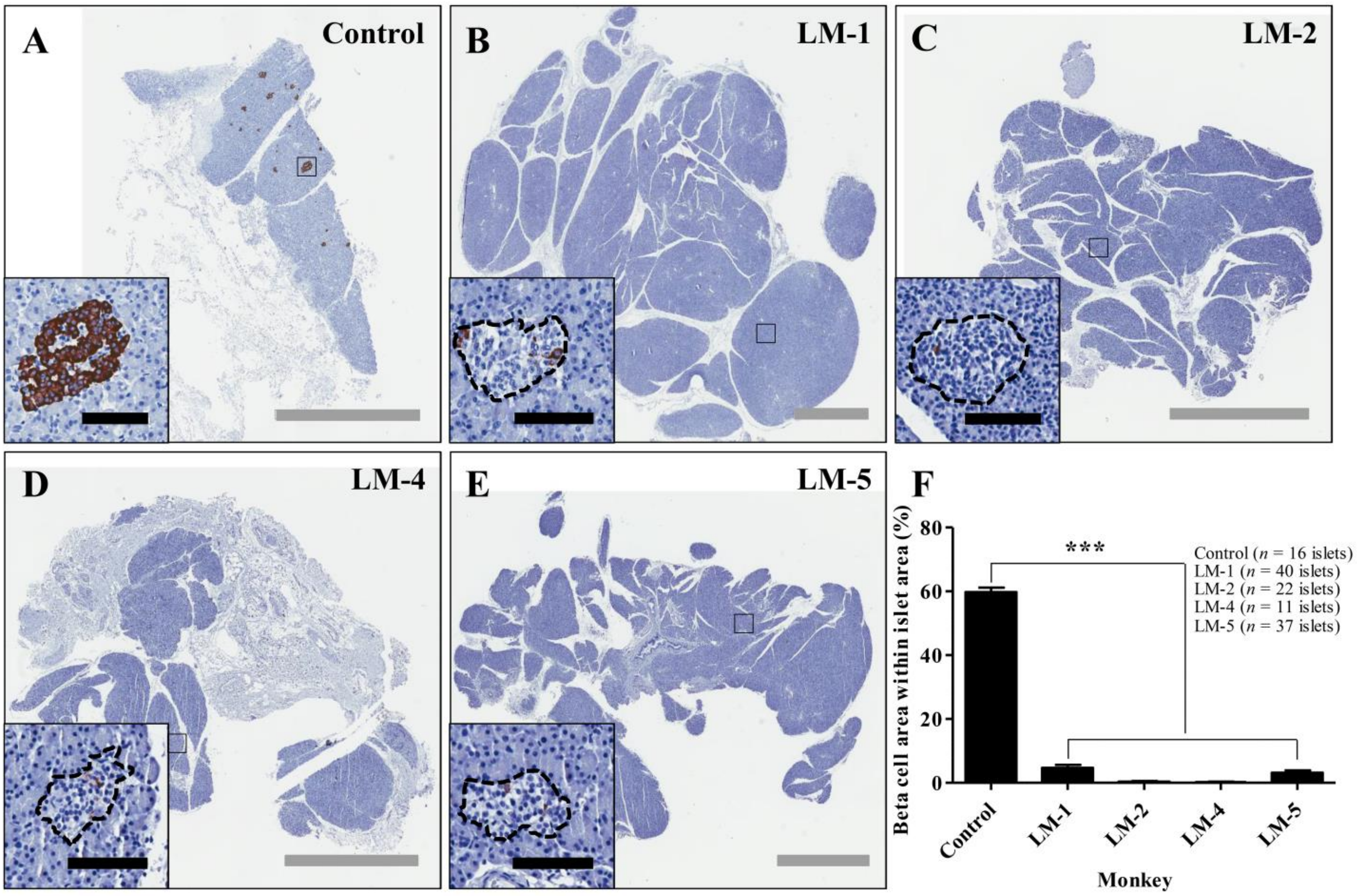

Supplemental Figure S2. Results of pancreas histology. In order to observe pancreatic islets, insulin staining was performed on the pancreas of monkeys LM-1, LM-2, LM-4, and LM-5 obtained at the end of the experiment and normal monkeys obtained after pancreatectomy. (a) Normal monkeys maintained intact islets, and beta cells expressed abundant insulin. In contrast, in monkeys (b) LM-1, (c) LM-2, (d) LM-4, and (e) LM-5, only islets replaced by scar tissue (black dotted lines) containing beta cells that rarely expressed insulin were observed. (f) The ratio of beta cells in islets was significantly different between normal monkeys and diabetic monkeys. Scale = bar, 100 μm (black), 2 mm (gray). ***$p < 0.001$.

Supplemental figure 3.

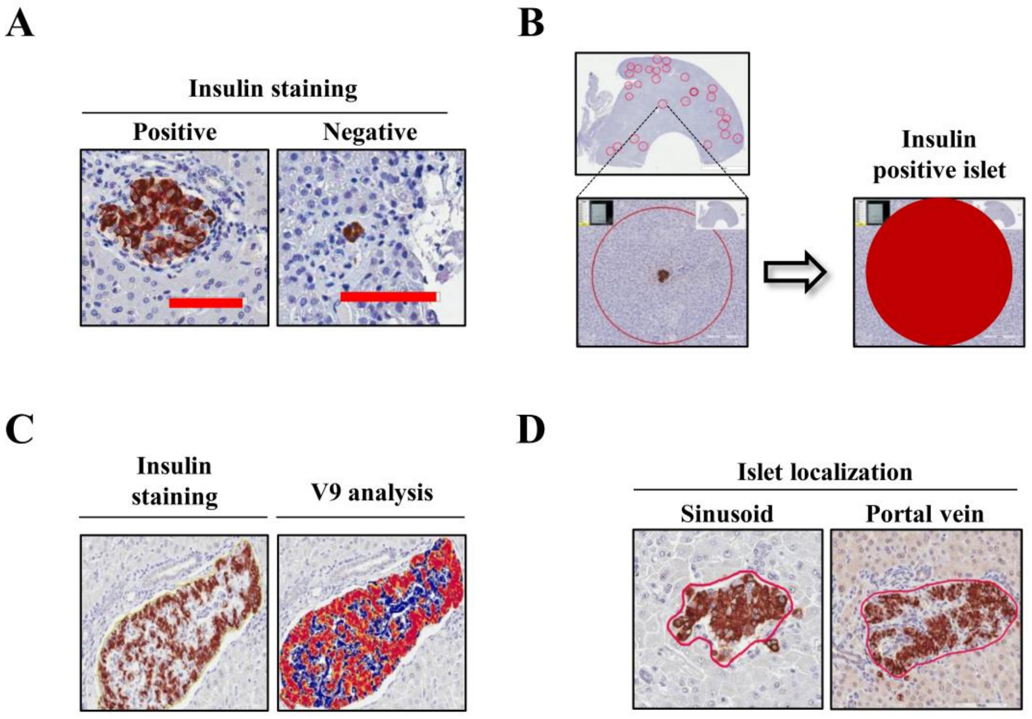

Supplemental Figure S3. The criteria for insulin-positive islets, beta cell area, and grafted location of islets. (**a**) Insulin-positive islets were based on a diameter >50 μm (scale bar = 50 μm). (**b**) To visualize transplanted islets in the whole liver, counted islets are indicated with red circles on analyzed liver tissue. (**c**) Counted islets and liver tissues were analyzed using the Positive Pixel Count algorithm v9.1 to obtain the perimeter and area. (**d**) The location of grafted islets was designated as portal vein or sinusoid.

Supplemental figure 4.

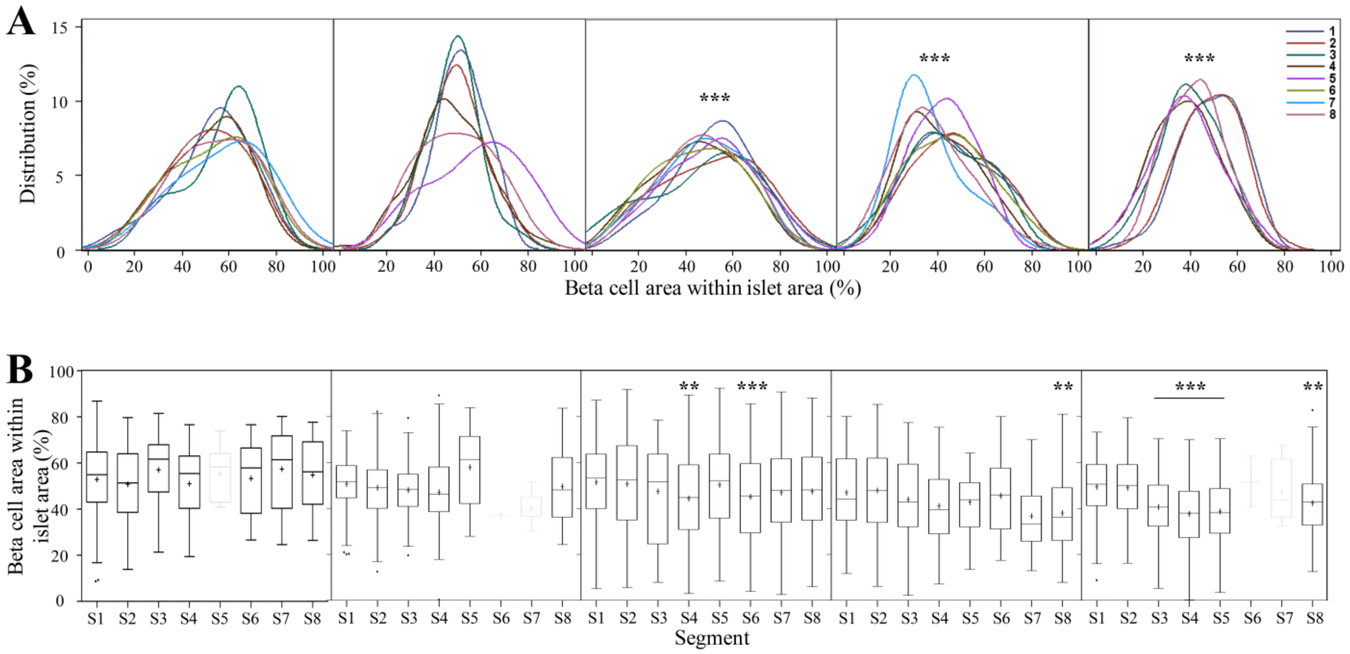

Supplemental Figure S4. The characteristics of transplanted islets in the five monkey livers were similar in whole segments. The beta cell area of grafted islets was obtained after analysis using the Positive Pixel Count algorithm. (**a**) Linear graphs represent the overall similarity between monkeys. (**b**) The statistical similarity of the column graph indicates the similarity of each segment to S1 or S8. Segments with less than 10 islets are shown in light gray and were excluded from statistical analysis. **p < 0.01, ***p < 0.001.

Supplemental figure 5.

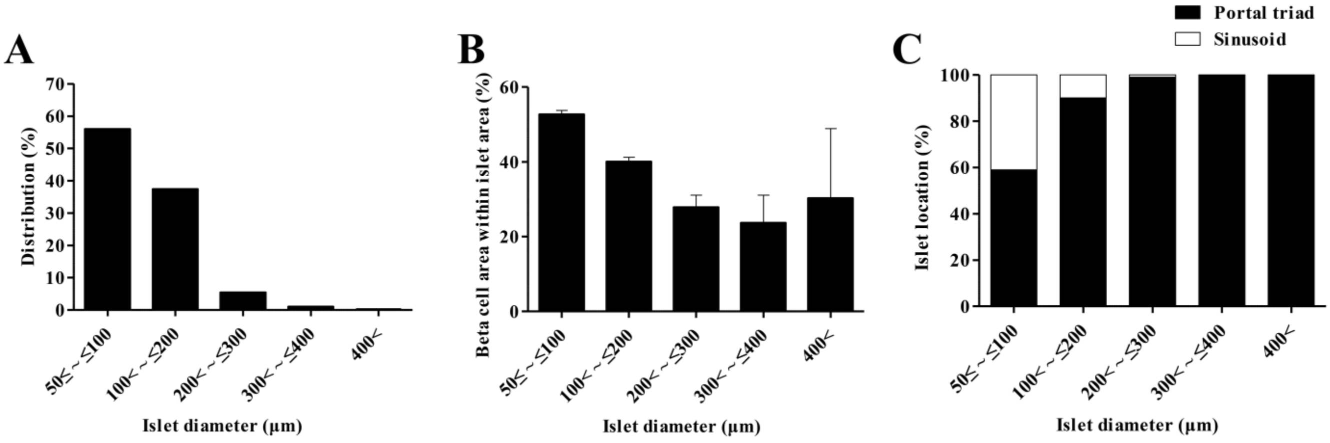

Supplemental Figure S5. The overall characteristics of islets in the five monkeys**.** (**a**) Islet diameter, (**b**) beta cell area of islet area, and (**c**) location of islets (n = 4,945 islets).
